# NADH oxidase-dependent CD39 expression by CD8^+^ T cells modulates interferon gamma responses via generation of adenosine

**DOI:** 10.1038/ncomms9819

**Published:** 2015-11-09

**Authors:** Aiping Bai, Alan Moss, Sonja Rothweiler, Maria Serena Longhi, Yan Wu, Wolfgang G. Junger, Simon C. Robson

**Affiliations:** 1 Division of Gastroenterology, Department of Medicine, Beth Israel Deaconess Medical Center, Harvard University, Boston, Massachusetts 02215, USA; 2 Department of Surgery, Beth Israel Deaconess Medical Center, Harvard University, Boston, Massachusetts 02215, USA

## Abstract

Interferon gamma (IFNγ)-producing CD8^+^ T cells (Tc1) play important roles in immunological disease. We now report that CD3/CD28-mediated stimulation of CD8^+^ T cells to generate Tc1 cells, not only increases IFNγ production but also boosts the generation of reactive oxygen species (ROS) and augments expression of CD39. Inhibition of NADPH oxidases or knockdown of gp91phox in CD8^+^ T cells abrogates ROS generation, which in turn modulates JNK and NFκB signalling with decreases in both IFNγ levels and CD39 expression. CD39^+^CD8^+^ T cells substantially inhibit IFNγ production by CD39^−^CD8^+^ T cells via the paracrine generation of adenosine, which is operational via adenosine type 2A receptors. Increases in numbers of CD39^+^CD8^+^ T cells and associated enhancements in ROS signal transduction are noted in cells from patients with Crohn's disease. Our findings provide insights into Tc1-mediated IFNγ responses and ROS generation and link these pathways to CD39/adenosine-mediated effects in immunological disease.

## Abstract (web summary)

The ectonucleotidase CD39 ultimately generates extracellular adenosine, modulating paracrine purinergic signaling. Here the authors show that IFNγ induction in CD8+ T cells is accompanied by NADH oxidase-dependent CD39 expression, which then inhibits IFNγ production in CD39-CD8+ T cells.

## Graphical abstract

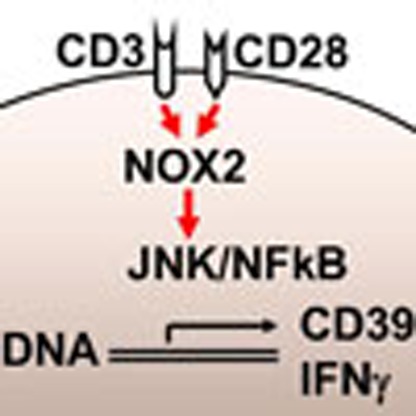

Adaptive immune cells, inclusive of CD4^+^ and CD8^+^ T cells, play important role in maintaining immune homeostasis. When perturbed, these cells become pathogenetic and release large amounts of proinflammatory cytokines, for example, interferon γ (IFNγ)^1^,^2^, which is recognized as one of the key inflammatory mediators in human immune diseases.

Crohn's disease, and other forms of inflammatory bowel disease, are chronic, immune-mediated intestinal disorders, characterized by excessive T-cell responses in genetically susceptible individuals^3^. Upon activation induced by luminal antigens, for example, from pathogenic bacteria, immune cells of patients with Crohn's disease produce substantial levels of proinflammatory cytokines including IFNγ, which further provoke inflammatory responses^4^,^5^. Indeed, IFNγ has multiple proinflammatory properties, that is, triggering epithelial apoptosis and barrier dysfunction, augmenting immune cell activation and inducing tissue damage^6^,^7^. Inhibiting IFNγ production has been shown to improve the symptoms of Crohn's disease^6^ and to decrease inflammatory markers in some studies^8^,^9^.

CD8^+^ T cells are one of the major adaptive immune cells. Type 1 CD8^+^ T cells (Tc1) have been reported to release high levels of IFNγ (ref. 10), and have been implicated in pathogen clearance, immune diseases and in antitumor immunity^11^,^12^. Recent data have shown that together with CD4^+^ T cells CD8^+^ T cells participate in immune responses of Crohn's disease^13^,^14^. Intriguingly, CD8^+^ T cells in Crohn's disease are also capable of producing substantial proinflammatory cytokines including IFNγ (ref. 13).

Reactive oxygen species (ROS) have been shown to modulate CD4^+^ T-cell function and proliferation^15^, which are likewise considered to be key factors in pathogenesis of immune diseases such as Crohn's disease^3^. Little is known as to how ROS might regulate CD8^+^ T-cell responses. Moreover, whether such cellular signals modulate IFNγ production of Tc1 cells in Crohn's disease remains largely unexplored.

Our prior studies indicate that murine experimental colitis is exacerbated by deletion of CD39 and further suggest that gene polymorphisms are associated with inflammatory bowel disease in humans^16^. CD39 (also termed ecto-nucleoside triphosphate diphosphohydrolase-1 or E-NTPDase1) is the dominant vascular and immune cell (for example, regulatory CD4^+^ T cell) ectonucleotidase, responsible for sequentially hydrolysing extracellular ATP and ADP to AMP; the latter is ultimately degraded to adenosine by CD73/ecto-5′-nucleotidase^17^,^18^. Adenosine is known to suppress immune responses through type 1 purinergic receptors, chiefly the adenosine type 2 A (A2A) receptor^19^,^20^. Recently, we have also noted that, in humans, CD39 expression in CD4^+^ T cells distinguishes regulatory T lymphocytes and other effector memory CD4^+^ T-cell populations. The latter cells, seemingly pathogenic or activated cell populations, have the capacity to secrete proinflammatory cytokines inclusive of IFNγ and interleukin (IL)-17 (refs 21, 22).

To date, the properties and functionality of CD39 on human CD8^+^ T cells and patterns of expression in immune diseases, such as Crohn's disease, have not been fully explored, and are therefore a further focus of this study. Here we demonstrate that CD39 labels those CD8^+^ T cells, which are high-level IFNγ-producing cells, and yet also exert suppressive functions. We also note that CD39 and IFNγ expression patterns in CD8^+^ T cells are regulated by CD3/CD28 signal cascades, inclusive of NADPH oxidases (NOX)/ROS, as well as downstream components of signalling involving c-Jun N-terminal kinase (JNK) and nuclear factor kappa B (NFκB). We further show that regulation of ROS signalling and heightened generation of adenosine can limit Tc1 effector cell responses, such as seen in Crohn's disease. We suggest that targeting IFNγ in inflammatory diseases might be achieved by modulation of both ROS signal and purinergic signalling in Tc1 cells.

## Results

### CD3/CD28-ROS signals determine Tc1 development

**Figure 1:**
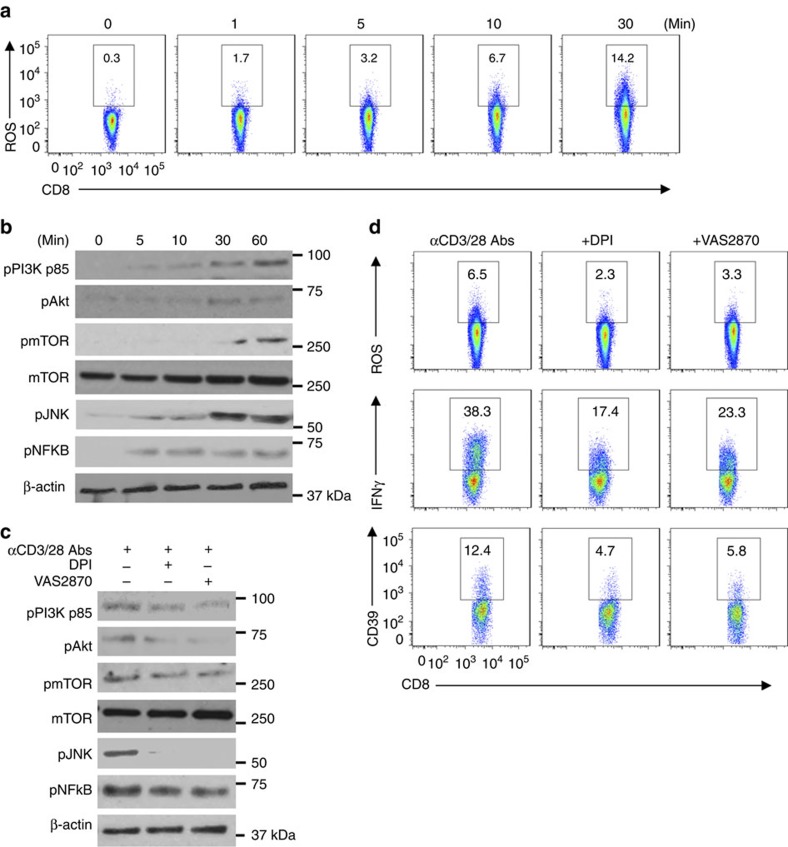
CD3/CD28-ROS signals modulate Tc1 development. (**a**,**b**) Representative fluorescence-activated cell sorting (FACS) analyses of ROS induction (**a**) and western blotting indicating phosphorylation of intracellular CD3/CD28 downstream signalling components (**b**) in healthy blood CD8^+^ T cells stimulated with anti-CD3/CD28 antibodies at different time points. Cells were pretreated with 4 μM of H_2_DCFDA to allow for ROS determination. (**c**,**d**) Healthy peripheral blood CD8^+^ T cells were stimulated with anti-CD3/CD28 antibodies in the presence or absence of DPI (10 μM) or VAS2870 (10 μM), both NOX inhibitors, followed by determination of CD3/CD28 signalling transduction at 60 min by western blot (**c**), ROS at 10 min and IFNγ or CD39 expression at 24 or 72 h by FACS, respectively (**d**). All data are representative of 3–4 independent experiments.

The importance and role of NOX/ROS signalling in functionalities of CD8^+^ T cells was first investigated. We noted that upon CD3/CD28 activation both production of ROS and phosphorylation of CD3/CD28 signalling components (including PI3K, Akt, mTOR, JNK and NFκB) gradually increased in CD8^+^ T cells in a time-dependent manner (Fig. 1a,b).

Furthermore, blockade of ROS signalling by NOX inhibitors diphenyleneiodonium chloride (DPI) and VAS2870 in these cells substantively dampened CD3/CD28 signalling transduction (Fig. 1c), concomitant with diminished ROS generation (Fig. 1d and Supplementary Fig. 1) and decreased IFNγ and CD39 expression (Fig. 1d). Meanwhile, it was noted that upon CD3/CD28 stimulation, CD8^+^ T cells expressed robust amounts of IFNγ, but minimal levels of IL-4, IL-17 and FOXP3/IL-10 (Supplementary Fig. 2). These data implicate NOX/ROS as a key upstream component of CD3 and CD28 signalling pathways controlling Tc1 cell development.

### CD39 expression in CD8^+^ T cells is JNK and NFκB dependent

**Figure 2:**
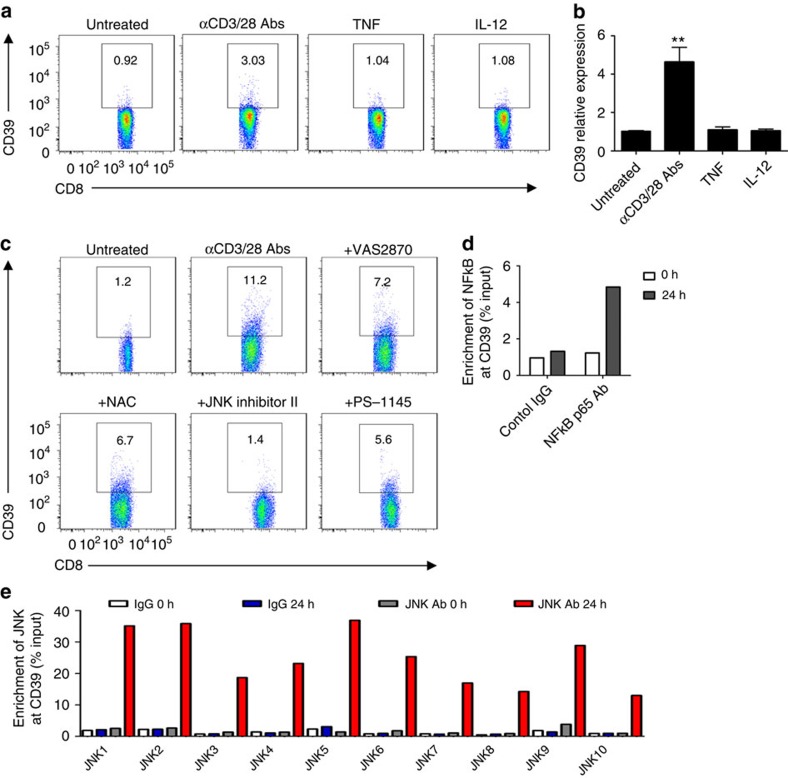
CD39 expression in CD8^+^ T cells is JNK and NFκB dependent. (**a**,**b**) Healthy blood CD8^+^ T cells were treated with anti-CD3 (10 μg ml^−1^, precoated) and anti-CD28 (5 μg ml^−1^, soluble) antibodies, or 10 ng ml^−1^ of the cytokines: either TNF or IL-12. CD39 expression was then determined by flow cytometry at 24 h (**a**) (*n*=4) or by quantitative PCR at 2 h (**b**) (*n*=4). (**c**) Healthy blood CD8^+^ T cells were stimulated with anti-CD3/CD28 antibodies in the presence or absence of VAS2870 (10 μM), NAC (10 mM), JNK inhibitor II (10 μM), PS-1145 (10 μM) for 72 h. CD39 expression was then analysed by fluorescence-activated cell sorting (*n*=3). (**d**,**e**) Chromatin immunoprecipitation analyses indicating enrichment of NFκB p65 (**d**) or JNK/c-Jun (**e**) at the CD39 promoter region in CD8^+^ T cells fresh isolated (fresh) or activated with anti-CD3/28 antibodies for 24 h (activated). Data are shown as mean±s.e.m., **P<0.001 (one-way analysis of variance), for the comparison with the other groups.

Next, we studied CD39 expression on differentially stimulated healthy CD8^+^ T cells *in vitro*. As determined by flow cytometry and quantitative real-time PCR (qRT–PCR), CD39 expression was induced by stimulation of CD8^+^ T cells with anti-CD3/CD28 antibodies, not by proinflammatory cytokines, for example, TNF and IL-12 (Fig. 2a,b and Supplementary Fig. 3a).

To further delineate CD3/CD28-NOX/ROS signalling in CD39 expression by CD8^+^ T cells, specific pharmacological inhibitors to the downstream components such as JNK and NFκB were employed in *in vitro* CD8^+^ T-cell culture systems. We noted that CD3/CD28-evoked CD39 induction was inhibited by either of these inhibitors, albeit with differential potency (Fig. 2c and Supplementary Fig. 3b).

It still remains unknown whether JNK and/or NFκB can directly drive CD39 expression. Sequence analyses of the promoter region of CD39 have identified a putative NFκB-binding site and up to 16 c-Jun/AP-1-binding sites (Supplementary Fig. 4). Chromatin immunoprecipitation analysis has shown that after activation both NFκB p65 and JNK (via directly binding to c-Jun/AP-1) are ‘preferentially' enriched at the promoter region of CD39 (Fig. 2d,e). These data infer that CD3/CD28-NOX/ROS signalling controls CD39 expression in CD8^+^ T cells through regulation of the transcriptional machinery of NFκB and JNK.

### CD39^+^CD8^+^ T cells exhibit Tc1 phenotypic responses

**Figure 3:**
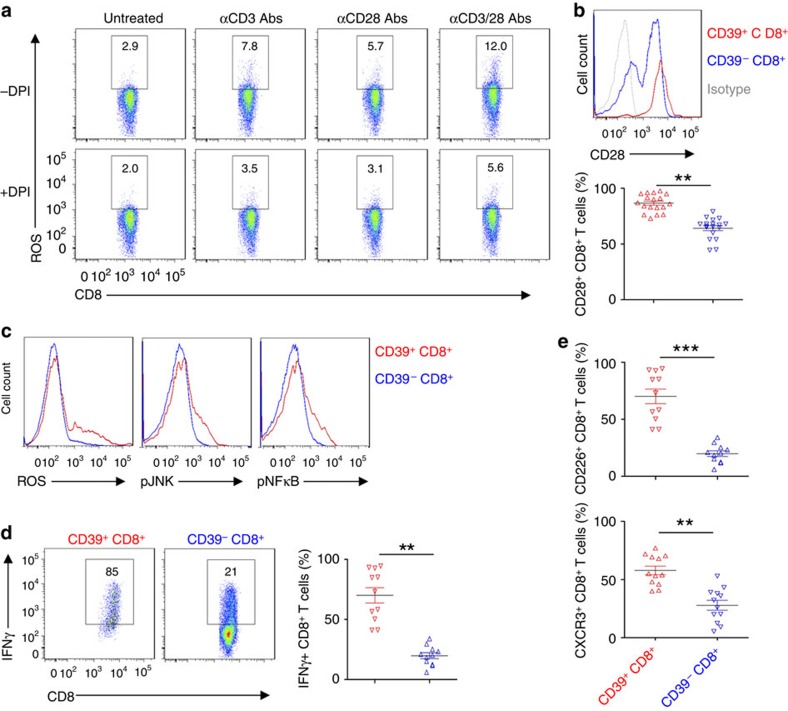
CD39^+^CD8^+^ T cells exhibit Tc1 responsiveness. (**a**) Healthy blood CD8^+^ T cells were stimulated with anti-CD3 (10 μg ml^−1^) or/and CD28 (5 μg ml^−1^) antibodies in the presence of vehicle or DPI (10 μM), and ROS induction was determined at 30 min. (**b**) Flow cytometric analyses of CD28 expression was done as based on expression of CD39 on CD8^+^ T cells (*n*=18); statistical analysis of percentages of two CD8^+^ T-cell subsets is shown in lower panel. (**c**) Flow cytometry of ROS induction, phospho-JNK and phospho-NFκB p65 in CD8^+^ T cells, stimulated with anti-CD3/CD28 antibodies for 30 min. Cells were pretreated with 4 μM of H_2_DCFDA for ROS determination, as before. (**d**) Representative flow cytometric analyses of CD8^+^ T cells based on expression of CD39. CD8^+^ T cells were stimulated with anti-CD3/CD28 antibodies for 24 h. Statistical comparative analysis indicating different percentages of CD8^+^ T cells expressing IFNγ is shown in the right panel. (**e**) Flow analysis of CD226 (*n*=11) and CXCR3 (*n*=12) expression on CD39^+^CD8^+^ and CD39^−^CD8^+^ T cells. Data are presented as means±s.e.m., **P<0.01, ***P<0.001 (Student's *t*-test).

Because CD3/CD28 stimulation triggered NOX/ROS signalling, we next sought to dissect out how CD3 and CD28 signals interact to modulate ROS signalling of CD8^+^ T cells. We noted that anti-CD3 and anti-CD28 antibodies exert strong synergistic effects on Tc1 responses, that is, ROS production (Fig. 3a), CD3/CD28 signal transduction (Supplementary Fig. 5a) and IFNγ production (Supplementary Fig. 5b). In parallel, all these CD3 and/or CD28 antibody-elicited Tc1 responses were markedly dampened by DPI (Fig. 3a and Supplementary Fig. 5). These results indicate that both CD3 and CD28 signals are indispensable for ROS-mediated activation of Tc1 cells.

We next contrasted CD3/CD28/ROS signals in healthy CD39^+^ and CD39^−^CD8^+^ T cells. Significantly higher levels of CD28 expression were observed in freshly isolated CD39^+^CD8^+^ T cells, when compared with CD39^−^CD8^+^ T cells from the same individual (Fig. 3b). Upon CD3/CD28 stimulation, CD39^+^CD8^+^ T cells exhibited heightened production of ROS and phosphorylation of JNK and NFκB, when compared with CD39^−^CD8^+^ T cells (Fig. 3c).

Next, we evaluated links between expression of CD39 and IFNγ on CD8^+^ T cells, and compared IFNγ productions by CD8^+^ T cells on the basis of CD39 expression. At 24 h post-stimulation with anti-CD3/CD28 antibodies, a significant proportion of CD39^+^CD8^+^ T cells was noted to be positive for IFNγ, when compared with CD39^−^CD8^+^ T cells (Fig. 3d). Meanwhile, the relative IFNγ-producing T-cell surface markers including CD226 and CXCR3 (refs 23, 24) were significantly greater in CD39^+^ CD8^+^ T cells, when compared with CD39^−^ CD8^+^ T cells (Fig. 3e).

Collectively, these data imply that CD39^+^CD8^+^ T cells exhibit Tc1 phenotype.

### CD3/CD28-ROS signals are associated with NOX2 bioactivity

**Figure 4:**
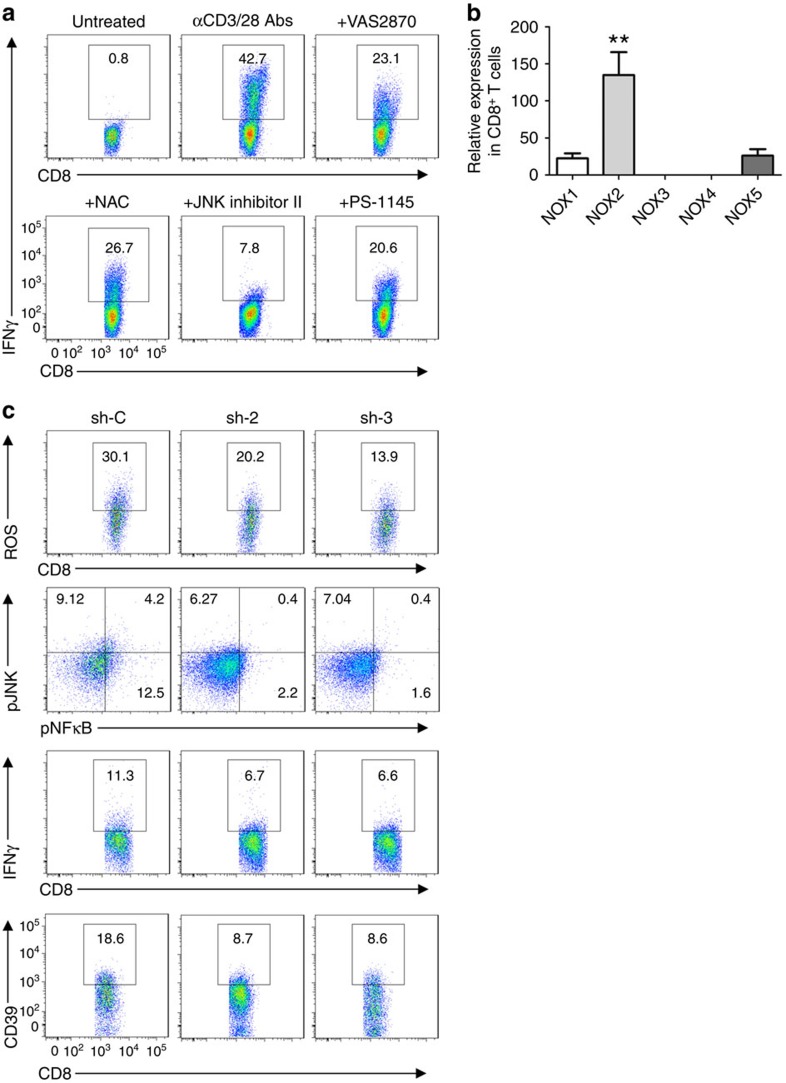
CD3/CD28-ROS signals are NOX2 dependent. (**a**) Representative flow cytometry of healthy blood CD8^+^ T cells, as determined on the basis of IFNγ expression (*n*=4). Freshly isolated CD8^+^ T cells were stimulated with anti-CD3 (10 μg ml^−1^, precoated) and anti-CD28 (5 μg ml^−1^, soluble) antibodies in the presence or absence of VAS2870 (10 μM), NAC (10 mM), JNK inhibitor II (10 μM) and PS-1145 (10 μM) for 24 h (*n*=3), as before. (**b**) Relative expression of NOX1–5 in healthy blood CD8^+^ T cells was determined by quantitative PCR, and GAPDH was used as internal control (*n*=3). (**c**) Control knockdown (sh-C) and NOX2 knockdown (sh-2 and sh-3) of healthy peripheral blood CD8^+^ T cells stimulated with anti-CD3/CD28 antibodies, followed by representative fluorescence-activated cell sorting analyses of: ROS at 60 min; CD3/CD28 signalling transduction pathways at 120 min and IFNγ or CD39 expression at 24 or 48 h, respectively. Cells were pretreated with 4 μM of H_2_DCFDA to allow for ROS examination. Data are presented as mean±s.e.m., **P<0.01 (one-way analysis of variance), with comparisons between groups.

It has been reported that JNK and NFκB are downstream components of CD3/CD28 signalling and have been implicated in the control of IFNγ expression^25^,^26^. We have employed specific pharmacological inhibitors to the downstream components such as JNK and NFκB, and studied Tc1 responses regulated by NOX/ROS signalling. We noted that CD3/CD28-evoked NOX/ROS signalling determined Tc1 responses, as shown by IFNγ expression and is abrogated by these inhibitors (Fig. 4a and Supplementary Fig. 6).

**Figure 5:**
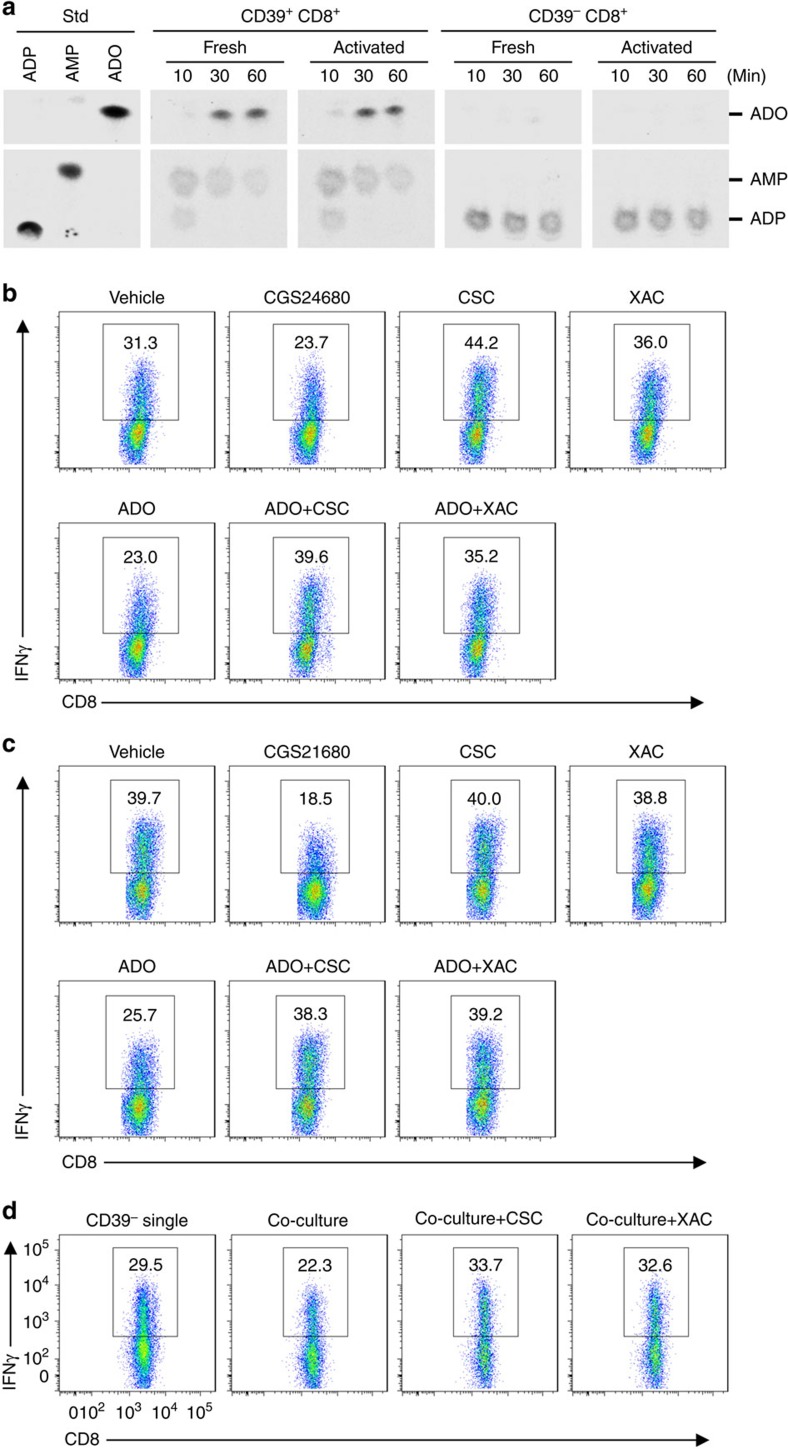
Purinergic signalling modulates Tc1 responses. (**a**) CD39^+^CD8^+^ or CD39^−^CD8^+^ T cells were used after purification (fresh) or after activation using anti-CD3/CD28 antibodies for 3 h (activated). Phosphohydrolysis of extracellular ^14^C-radiolabelled ADP catalysed by fresh or activated CD39^+^CD8^+^ or CD39^−^CD8^+^ T-cell subsets was determined by TLC. (**b**,**c**) Total CD8^+^ T cells (**b**) or freshly sorted CD39^−^CD8^+^ T cells (**c**) were stimulated with anti-CD3/CD28 antibodies in the presence of vehicle or CGS21680 (100 nM), adenosine (ADO, 50 μM) alone or together with CSC (500 nM) or XAC (1 μM) for 24 h, IFNγ expression was analysed by fluorescence-activated cell sorting (FACS). (**d**) Carboxyfluorescein succinimidyl ester-labelled CD39^−^CD8^+^ T cells alone or co-cultured with CD4^+^CD39^+^CD161^+^ T cells were stimulated with anti-CD3/CD28 antibodies for 24 h followed by FACS analysis of IFNγ expression. Co-cultures were incubated in the presence of vehicle, CSC (500 nM) or XAC (1 μM). Data are representative of three independent experiments.

NOX are a group of membrane-bound enzyme complexes regulating ROS generation, composed of several subunits^27^. The quantitative PCR analysis indicated that human CD8^+^ T cells preferentially expressed gp91phox (NOX2) (Fig. 5b). We have employed two efficient lentiviral small hairpin RNA (shRNA) sequences to knockdown NOX2 (Supplementary Fig. 7), and further demonstrated that knockdown of NOX2 in CD8^+^ T cells result in decreased ROS production as well as decreased levels of JNK and NFκB phosphorylation (Fig. 4c). These mechanisms have impacts on both IFNγ and CD39 expression (Fig. 4c), indicating the control of Tc1 development and CD39 expression by NOX2-dependent CD3/CD28-ROS-mediated signals.

### Purinergic signalling modulates Tc1 responses

We have previously demonstrated that extracellular purinergic signalling, specifically by adenosine generated by the ectonucleotidases CD39 and CD73, impacts CD4^+^ T-cell function^17^ and modulates adaptive immune responses^28^. Conversion of extracellular ATP ultimately to adenosine has also been shown to inhibit CD8^+^ T-cell activation and cytokine production via A2A receptor responses^29^,^30^. To study this phenomenon further, we have investigated the specific impact of purinergic signalling on Tc1 induction.

We initially determined the expression patterns of plasma membrane CD73 and adenosine deaminase (ADA) on CD8^+^ T cells on the basis of CD39 expression. CD73 levels in both CD39^+^ and CD39^−^CD8^+^ T-cell subsets were similar (Supplementary Fig. 8a). However, ADA was specifically expressed by CD39^+^CD8^+^ T cells (Supplementary Fig. 8b), indicating the capacity of CD39^+^CD8^+^ T cells to degrade locally generated adenosine into the derivative product inosine^31^.

NTPDase enzymatic activity of CD39^+^ and CD39^−^CD8^+^ T cells was examined by studying hydrolysis of ^14^C-radiolabelled nucleotides. Substantive ectonucleotidase activity was noted in CD39^+^CD8^+^ T cells, which efficiently catalyse the conversion of ADP to adenosine, regardless of the cell activation status (Fig. 5a). In contrast, CD39^−^CD8^+^ T cells did not exhibit relevant NTPDase activity or generate extracellular adenosine (Fig. 5a). These data infer that CD39^+^CD8^+^ T cells are the major source of extracellular adenosine among total CD8^+^ T cells.

Next, we determined Tc1 responses after exposure of healthy blood CD8^+^ T cells to various adenosine receptor agonists and antagonists. As shown in Fig. 5b and Supplementary Fig. 9a, CGS21680 or adenosine, two exogenous agonists (at the A2A receptor), diminished IFNγ levels, while 8-(3-chlorostyryl) caffeine (CSC, a specific A2A antagonist) or xanthine amine congener (XAC, a pan adenosine receptor antagonist) restored IFNγ production generated by the CD8^+^ T cells. Furthermore, combinations of adenosine with CSC or XAC completely rescued IFNγ-producing capacity of the total CD8^+^ T cells that had been previously inhibited by adenosine (Fig. 5b).

We also extended these adenosine receptor studies in sorted CD39^−^CD8^+^ T cells. IFNγ production by CD39^−^CD8^+^ T cells recapitulated the patterns of total CD8^+^ T cells in response to A2A agonist treatment (Fig. 5c and Supplementary Fig. 9b). However, either CSC or XAC alone had minimal effects on Tc1 responses in CD39^−^CD8^+^ T cells (Fig. 5c).

The functional interactions between CD39^+^ and CD39^−^CD8^+^ T cells were then examined. IFNγ-producing capacity of CD39^−^CD8^+^ T cells was diminished in the presence of co-cultured CD39^+^CD8^+^ T cells, but the inhibition induced by CD39^+^CD8^+^ T cells was completely reversed by co-treatment with CSC or XAC (Fig. 5d and Supplementary Fig. 9c). These results are indicative of the involvement of A2A receptor signalling in the paracrine-type inhibition of the CD39^+^CD8^+^ T cells on CD39^−^CD8^+^ T cells.

### CD39^+^CD8^+^ T cells are increased in Crohn's disease

In immunologically mediated diseases such as Crohn's disease, CD8^+^ T-cell activation can be boosted by activated antigen-presenting cells and this occurs, at least in part, via elicitation of CD3/CD28 signalling^32^. We therefore evaluated patterns of CD39 expression by CD8^+^ T cells in patients with Crohn's disease under basal conditions and after cell activation.

**Figure 6:**
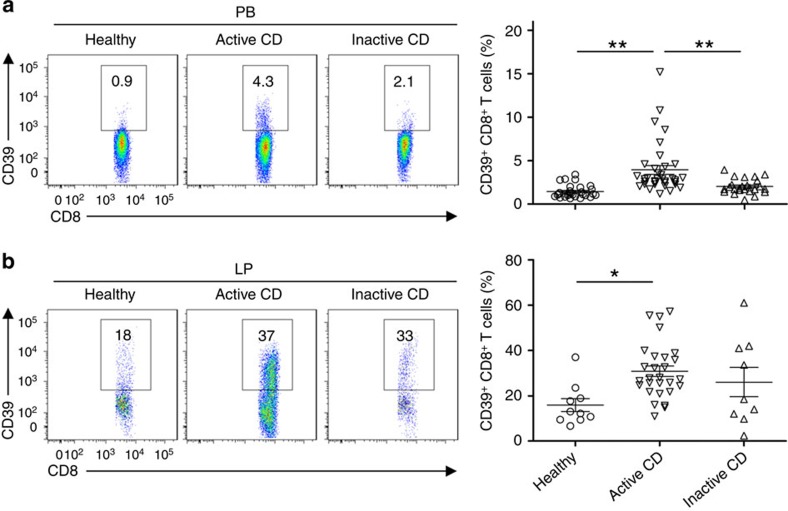
CD39^+^CD8^+^ T cell numbers are increased in Crohn's disease (CD). (**a**,**b**) Flow cytometric analyses of CD39 expression on CD8^+^ T cells in peripheral blood (PB) (**a**) or lamina propria (LP) (**b**) of healthy volunteers, patients with active and inactive CD (*n*=29, 35 or 25 for peripheral blood; 10, 28 or 9 for lamina propria, respectively). Numbers in quadrants indicate percentage of the cells in the designated gates. Data are presented as means±s.e.m., *P<0.01, **P<0.001 (one-way analysis of variance).

As shown in Fig. 6a, the percentage of CD39^+^ CD8^+^ T cells was significantly increased in peripheral blood of patients with active Crohn's disease (3.9±0.5%), as compared with healthy donors (1.44±0.1%) or with those who had inactive disease (2.0±0.5%). In parallel, lamina propria levels of these CD39^+^CD8^+^ T cells in active and inactive Crohn's disease patients (29.6±2.9% and 24.9±4.4%) were substantially higher than that in healthy controls (13.1±3.1%; Fig. 6b).

### CD8^+^ T cells in Crohn's disease exhibit ROS signalling

**Figure 7:**
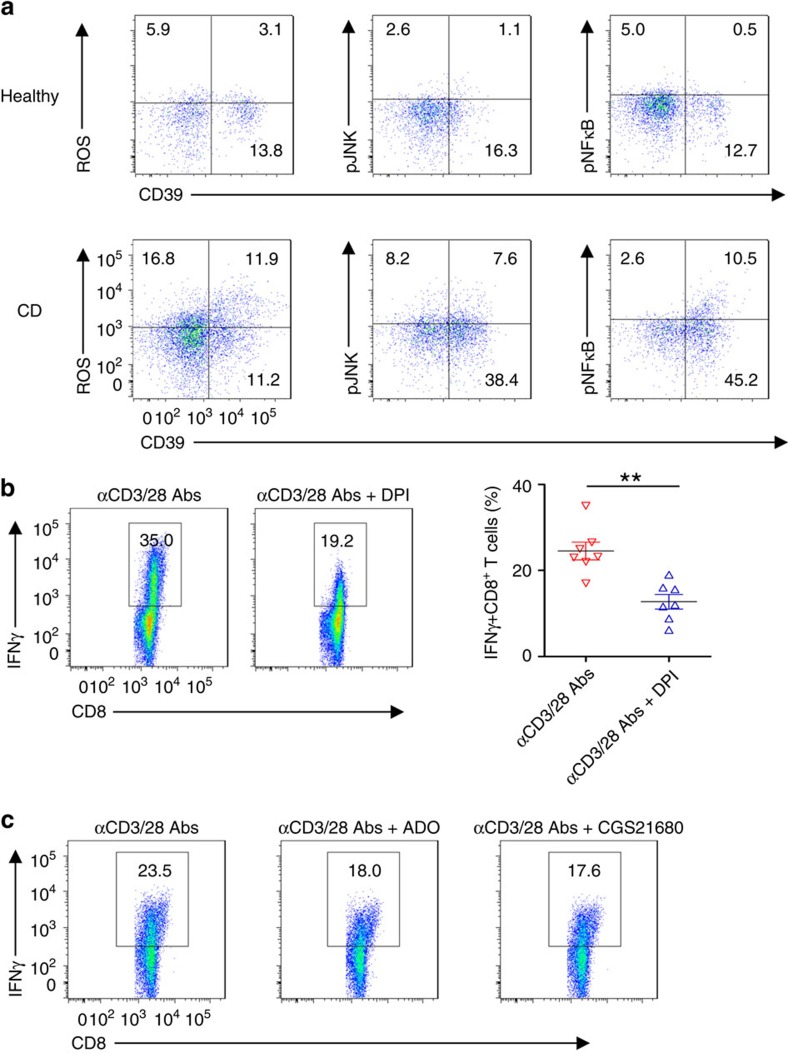
CD8^+^ T cells in Crohn's disease (CD) exhibit ROS signalling. (**a**) Representative fluorescence-activated cell sorting (FACS) analyses of ROS induction and intracellular pJNK and pNFkB levels in lamina propria (LP) CD8^+^ T cells of healthy donors and active CD patients. LP CD8^+^ T cells were stimulated with anti-CD3/CD28 antibodies for 30 min. Cells were pretreated with 4 μM of H_2_DCFDA for ROS examination. Data are representative of 4–5 independent experiments. (**b**) Peripheral blood CD3^+^CD8^+^ T cells isolated from patients with active CD were stimulated with anti-CD3/CD28 antibodies in the presence of vehicle or DPI (10 μM) for 24 h, followed by determination of intracellular IFNγ levels by FACS. Statistical analyses of percentages of Tc1 are shown on the right in each panel (*n*=7). Data are shown as means±s.e.m., **P<0.005 (Student's *t*-test). (**c**) Peripheral blood CD8^+^ T cells of active CD patients were stimulated with anti-CD3/CD28 antibodies in the presence of vehicle, adenosine (ADO, 50 μM) or CGS21680 (100 nM), followed by analyses of IFNγ expression at 24 h by FACS. Data are representative of three independent experiments.

Recently, ROS have been shown to regulate immune cell function including CD8^+^ T cells^33^, and such mediators are generated in large amounts at sites of tissue inflammation, as in Crohn's disease^34^. We hypothesized that NOX/ROS signalling would determine Tc1 development, and therefore evaluated the expression of ROS signalling in CD8^+^ T cells of active Crohn's disease. We noted that levels of CD8^+^ T cells expressing ROS, phosphorylated JNK and NFκB p65 were substantially higher in lamina propria of active Crohn's disease patients, when compared with those cells obtained from healthy controls (Fig. 7a and Supplementary Fig. 10).

Intriguingly, blockade of NOX/ROS by DPI markedly abrogated IFNγ production in blood (Fig. 7b) and lamina propria (Supplementary Fig. 11a) CD8^+^ T cells obtained from patients with active Crohn's disease.

These data suggest a pivotal role of NOX/ROS signalling in Tc1 generation and in mediating the associated inflammatory responses seen in Crohn's disease.

Next, to evaluate the role of inhibitory A2A signalling in the regulation of Tc1 responses, we treated CD8^+^ T cells of active Crohn's disease patients with A2A receptor agonists. As shown in Fig. 7c and Supplementary Figs 11b,12, A2A receptor signalling following use of adenosine or/and more specific agonists, for example, CGS21680, decreased IFNγ production of both blood and lamina propria CD8^+^ T cells from Crohn's disease patients *in vitro*. These last data suggest that immune regulation of Tc1 responses occurs via purinergic signalling, and is modulated, in particular, via A2A receptor.

## Discussion

IFNγ is one of the key inflammatory cytokines that mediate immune responses in human inflammatory disease. In the present study, the population of CD39 expressing CD8^+^ T cells in healthy controls, as well as those in Crohn's disease, can be shown to produce preferentially greater levels of IFNγ on a per-cell basis. These cells exhibit both phenotypic and functional characteristics of Tc1 cells^35^. We note that polyclonal activation of CD8^+^ T cells (by anti-CD3/CD28 antibodies) boosts CD39 expression on these cells, consistent with the increased frequency of CD39^+^CD8^+^ T cells seen in the blood and inflamed tissues of patients with active Crohn's disease. We suggest that pretreatment evaluation of CD39^+^CD8^+^ T cells might provide insights into the mechanisms through which clinical responses to anti-IFNγ and other immunomodulatory therapies could be determined^36^.

We show that stimulation of human CD8^+^ T cells with both anti-CD3 and CD28 antibodies, can induce ROS generation and enhance downstream signalling cascades. Indeed, these activation responses exert synergistic effects on ROS signalling, IFNγ production and CD39 expression. We also show that ROS generation and CD3 and CD28 intracellular signal cascades, inclusive of JNK and NFκB, are modulated by NOX2 in human CD8^+^ T cells.

Recently, there have been reports on the regulation of CD4^+^ T-cell function and proliferation by ROS^15^. These ROS mediators comprise reactive oxygen molecules, which are important in the regulation of cell signalling and function, particularly in the instance of immune cells^37^. Within cells, ROS are generated by transfer reaction of electrons through the mitochondrial respiratory chain, in part initiated by activation of NOX. There is, however, extensive crosstalk involving other cellular systems that generate ROS, such as endothelial nitric oxide synthase and xanthine oxidase^26^ as well as others, inclusive of lipoxygenases^38^,^39^,^40^. These systems all act in conjunction with mitochondrial electron transfer reactions to both bolster and modulate cellular ROS generation^38^,^39^,^40^. Once generated via NOX and mitochondrial electron transport chains^41^,^42^, ROS play important roles in mediating cell signalling responses^26^,^42^, and thereby impact a wide variety of physiological and pathological processes^43^,^44^.

In T cells, the NOX multicomponent electron transferase systems that use cytoplasmic NADPH to convert molecular oxygen to superoxide anions, appear largely responsible for cellular ROS generation^42^, rather than other ROS generating enzymes. In this present study, we have found that human CD8^+^ T cells preferentially express gp91phox (NOX2). Moreover, inhibition of NOX by inhibitors or knockdown of gp91phox (NOX2) abrogate ROS production, further indicating the pivotal role of NOX2 in ROS generation in human CD8^+^ T cells. Specifically, the lipoxygenase systems, as mentioned above, appear to have limited effects in these studies, as shown by others^42^.

However, the exact role of ROS mediators on T-cell function remains unclear. For example, these ROS have been reported to contribute significantly to the dominant T-helper effector phenotype and IL-17 production in autoimmunity^45^. In contrast, other researchers have shown that NOX and ROS deficiency results in skewed Th17 (ref. 46) and indeterminate Th1 responses^47^ in murine experimental models. Recently, the inhibition of NOX activity has been reported to ameliorate influenza virus-induced lung inflammation^48^, suggesting association between NOX/ROS and CD8^+^ T-cell function.

Mechanistically, on stimulation, we propose that NOX2/ROS signalling modulates CD3/CD28 intracellular signal cascades. JNK and NFκB, as downstream signal components of CD3/CD28 activation and other key putative elements, control IFNγ production^25^,^26^, as well as CD39 expression. These pathways appear to involve regulation of JNK and NFκB responses in CD8^+^ T cells, resulting in Tc1 deviation and heighted CD39 expression, which subsequently induce extracellular ATP/ADP phosphohydrolysis to impact purinergic signalling.

We also show that CD39, a prominent immune cell-expressed ectonucleotidase^49^, provides CD8^+^ T cells with the capacity to project local inhibitory functionality. The increased levels of CD39 induced by stimulation of anti-CD3 and CD28 antibodies boost CD8^+^ T cell-mediated hydrolysis of ATP/ADP, in tandem with CD73 (ecto-5′-ectonucleotidase), to generate exogenous adenosine. We have previously shown that such purinergic signalling effects, that is, through generation of extracellular adenosine, limits CD4^+^ T-effector cell immune responses^17^. This inhibitory effect has been further confirmed in CD8^+^ T cells to be also operational via A2A receptor responses^29^,^50^.

We have evaluated the role of CD39 expression in regulation of IFNγ production of CD8^+^ T cells. We note that CD39^+^CD8^+^ T cells are the major source of adenosine generation, and decrease IFNγ production of other CD8^+^ T cells, in a paracrine manner, at least in part, through the regulation of adenosine/A2A signalling responses. These latter pathways have been demonstrated in CD8^+^ T cells^29^,^50^, and appear comparable to the signal regulation pathways previously mapped in CD4^+^ T cells^51^,^52^.

**Figure 8:**
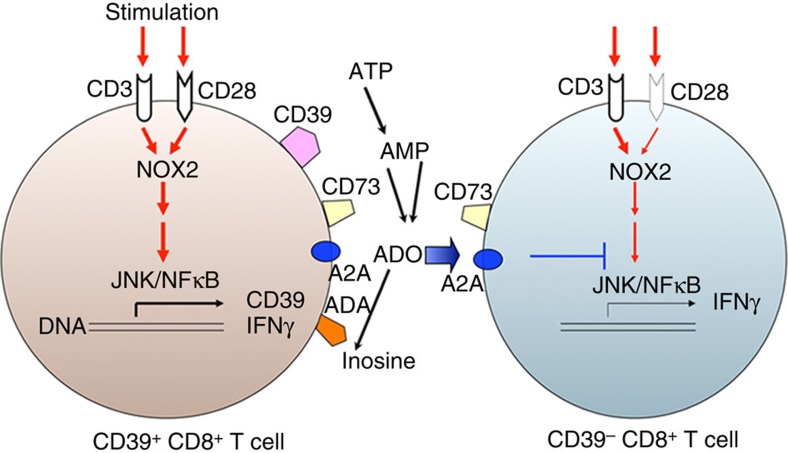
Schematic illustration of role of CD39 in Tc1 biology. Anti-CD3 or/and CD28 stimulation induces ROS generation, which is associated with the activation of CD3 or/and CD28 intracellular signaling cascades, induction of IFNγ production and heightened CD39 expression in CD8^+^ T cells. Because of preferential CD28 expression, CD39^+^CD8^+^ T cells exhibit prominent ROS signalling and show excessive IFNγ production. CD39^+^CD8^+^ T cells also initiate purinergic signalling and generate adenosine, which can further inhibit JNK and NFκB signalling and decrease IFNγ production by these CD39^−^CD8^+^ T cells via A2A receptor responses^51^,^52^.

We propose that the expression of CD39 in CD8^+^ T cells is induced by T-cell receptor stimulation (anti-CD3 or in combination with anti-CD28 antibodies). In this current study, we illustrate a possible schema of Tc1 responsiveness that could be regulated by CD39 expression on CD8^+^ T cells (Fig. 8). We propose that upon anti-CD3 or/and CD28 antibody stimulation, ROS generation and the linked activation of intracellular signal cascades can be rapidly initiated. This process is followed by induction of IFNγ production and the associated upregulation of CD39 plasma membrane expression on CD8^+^ T cells. Following preferential CD28 expression and signalling, CD39^+^CD8^+^ T cells show enhanced ROS generation with augmented activation of signal cascades, involving both JNK and NFκB.

These CD39^+^CD8^+^ T cells also initiate purinergic signalling and generate adenosine, which in turn can further diminish JNK and NFκB signalling and IFNγ production of CD39^−^CD8^+^ T cells via A2A receptor^51^,^52^. We also propose that the specific cell-associated expression of ADA might rescue CD39^+^CD8^+^ T cells from adenosine-induced inhibition of intrinsic Tc1 responses (Fig. 8). As a consequence of these complex signalling pathways, CD39^+^CD8^+^ T cells are able to limit their own intrinsic responsiveness to adenosine. Such cells could preferentially develop into IFNγ-producing cells in human immune diseases, such as Crohn's disease.

Finally, we demonstrate that the inhibition of ROS generation by DPI, an inhibitor of NOX, abrogates Tc1 response of CD8^+^ T cells in the blood and gut tissues of Crohn's disease. As a group of membrane-bound oxidases, NOX are responsible for ROS generation in a wide variety of cells and participate in regulation of cell activation and tissue function^53^,^54^. However, overactivation of NOX promotes excessive ROS generation and numerous pathological responses, and has been linked with human diseases such as granulomatous disease and cerebrovascular disease^55^,^56^. We can show that treatment with NOX inhibitor blocks IFNγ production and diminishes Tc1 responses in Crohn's disease, perhaps indicating that inhibition of NOX2/ROS signalling could be further explored as a potential therapeutic target.

In summary, we show that CD39 can be used to phenotypically define certain CD8^+^ IFNγ-producing cells. In addition, Tc1 responses by such cells expressing CD39 can be abrogated by inhibition of ROS signals. CD39^+^CD8^+^ Tc1 cells also limit IFNγ production of CD39^−^CD8^+^ T cells by generating adenosine that acts in a paracrine manner. These studies infer that strategies to regulate purinergic signalling and boost adenosine generation might decrease Tc1 responses in patients with IFNγ-dominant inflammatory diseases, such as seen in Crohn's disease.

## Methods

### Cells

Peripheral blood CD8^+^ T cells from healthy volunteers or patients with Crohn's disease were isolated using Human CD8^+^ T cell Enrichment Kit or Cocktail kit (both negative selection, from StemCell Technologies, Vancouver, Canada) according to the instruction with minor modification. Purities of CD3^+^CD8^+^ T cells isolated with the two kits above were >95%, as determined using FACSaria cell sorter (BD Biosciences, San Jose, CA, USA).

### *In vitro* CD8^+^ T-cell culture and stimulation

Isolated CD3^+^CD8^+^ T cells were cultured in complete RPMI 1640 medium (Invitrogen, Carlsbad, CA, USA) supplemented with 2 mM L-glutamine, 100 U ml^−1^ penicillin, 100 μg ml^−1^ streptomycin, 1% non-essential amino acids and 10% fetal calf serum. For stimulation, 5 × 10^5^ ml^−1^ T cells were treated with coated anti-CD3 antibody (OKT3, BioLegend, San Diego, CA, USA; 10 μg ml^−1^) and soluble anti-CD28 antibody (CD28.2, BioLegend, San Diego, CA, USA; 5 μg ml^−1^) for indicated time.

For co-culture studies, 5 × 10^5^ ml^−1^ CD39^−^CD8^+^ T cells were prestained with carboxyfluorescein succinimidyl ester (Invitrogen, Carlsbad, CA, USA; 2.5 μM) and cultured alone or together with equal number of CD39^+^CD8^+^ T cells in the presence of anti-CD3/CD28 antibodies. The reagents or control vehicle were introduced to cell cultures at the beginning of stimulation.

### Patients

All human studies were conducted in accordance with the Declaration of Helsinki and were approved by the BIDMC Institutional Review Committee (No. 2011-P-000202/8).

Patients with Crohn's disease (57 male and 40 female; age range, 19–71 years; who had ileocolonic or colonic disease) were recruited at BIDMC during routine clinic visits. Amongst those patients, 25 had either received in the recent past or were still receiving anti-TNF treatment; 11 were being treated with oral steroids, 10 had immunosuppressive treatments (inclusive of azathioprine or 6-MP) and 24 received combination treatment (5 with anti-TNF treatment and oral steroids, 15 with anti-TNF treatment and azathioprine/6-MP, 1 with oral steroids and azathioprine, and 3 with those three drugs), while the other 27 patients were studied at the initial presentation. Informed consent was obtained in writing from all enrollees. The diagnosis of Crohn's disease was confirmed on the basis of clinical, radiological, endoscopic and histological criteria. Peripheral blood was obtained during clinical blood draws and tissue biopsies during colonoscopies for disease staging surveillance.

### General reagents

All chemicals were purchased from Sigma-Aldrich (St Louis, MO, USA), unless otherwise stated. All cell culture media and reagents were from Invitrogen.

All cytokines were from R&D systems (Minneapolis, MN, USA).

### Antibodies

Fluorescence-activated cell sorting studies were performed using: FITC-, PE-, PE-Cy5, APC-Cy7, PE-Cy7, Pacific blue-, or APC-conjugated anti-human antibodies to: CD3 (clone#: HIT3a, 1:50), CD8 (SK1 or RPA-T8, 1:20), IL-17 (BL168, 1:20), IFNγ (4 S.B3, 1:10), IL-10 (JES3-19F1, 1:10), IL-4 (8D4-8, 1:10), FOXP3 (206D, 1:10), CD39 (A1, 1:20), CD73 (AD2, 1:20), CD28 (CD28.2, 1:20), CD45RA (HI100, 1:20), CD226 (TX25, 1:20), CXCR3 (G025H7, 1:20), Tim3 (F38-2E2, 1:20), CCR6 (G034E3, 1:20), Perforin (B-D48, 1:20), Granzyme B (GB11, 1:20) and CCR7 (g043h7, 1:20) from BioLegend (San Diego, CA, USA); CD39 (BU61, 5 μg ml^−1^) from Ancell Corporation (Bayport, MN, USA); Annexin V from BD Biosciences (1:20; Franklin Lakes, NJ, USA); phospho-JNK (Thr183/Tyr185; G9, 1:20) and phospho-NFκB p65 (Ser536; 93H1, 1:20) from Cell Signalling Technology (Danvers, MA, USA); and Isotype control antibodies from Ancell Corporation or eBioscience (San Diego, CA, USA).

Antibodies used for western blot included the following: phospho-PI3K p85 (Tyr458; #4228, 1:800), phospho-Akt (Ser473; #9271, 1:1,000), phospho-mTOR (Ser2448; #2971, 1:1,000), mTOR (#2972, 1:1,000), pJNK (Thr183/Tyr185; #9251, 1:1,000) and phospho-NFκB p65 (Ser536; #3031, 1:1,000), from Cell Signaling Technology (Danvers, MA, USA); NOX2 (ab31092, 1 μg ml^−1^) and β-actin (AC-15, #ab6276, 1:40,000) from Abcam (Cambridge, MA, USA).

### Purification of lamina propria mononuclear cells

Lamina propria mononuclear cells were isolated from freshly biopsied colonic mucosa^57^,^58^. Briefly, tissues were incubated in HBSS containing EDTA (0.75 mM) and dithiothreitol (1 mM) at 37 °C to remove the epithelium, followed by incubation with digestion cocktails (RPMI 1640 medium containing collagenase IV (400 U ml^−1^) and DNase I (0.01 mg ml^−1^)) at 37 °C. The digested tissues were filtered through a 100-μm cell strainer and centrifuged at 1,500 r.p.m. for 5 min. Cell pellets were resuspended in complete RPMI 1640 media and overlaid on a 40–100% Ficoll gradient (GE Healthcare Life Sciences, Pittsburgh, PA, USA). After centrifugation, the interphase (lamina propria mononuclear cells) was collected and washed once for subsequent experimentations.

### Western blotting

The cells were lysed on ice in modified RIPA buffer (50 mM Tris-HCl, pH 7.4; 1% NP-40; 0.25% sodium deoxycholate; 150 mM NaCl) supplemented with Complete Proteinase Inhibitor Cocktails (Roche Diagnostics, Indianapolis, IN, USA) and Phosphatase Inhibitor Cocktails (Sigma-Aldrich, St Louis, MO, USA). The lysates were spun at 10 000*g* for 5 min at 4 °C.

Protein concentrations were determined by Bio-Rad *DC* protein assay reagent (Bio-Rad Laboratories, Hercules, CA, USA). For western blot, 20 μg protein of each sample was separated on 4–12% Criterion XT Bis-Tris SDS-PAGE gels (Bio-Rad Laboratories, Hercules, CA, USA) and transferred to polyvinylidene difluoride membrane (Cat#IPVH00010, Millipore, Billerica, MA, USA) by semi-dry electroblotting. The latter were then probed with specific antibodies against proteins of interest. Bands were visualized using horseradish peroxidase-conjugated goat anti-mouse, donkey anti-rabbit, or donkey anti-sheep IgG (Thermo Scientific, Waltham, MA, USA) and the SuperSignal West Femto Maximum Sensitivity Substrate reagents (Cat#PI-34096, Thermo Scientific, Rockford, IL, USA) according to the manufacturer's instructions. Images have been cropped for presentation. Full-size images are presented in Supplementary Fig. 13.

### Chromatin immunoprecipitation

Chromatin was extracted from healthy CD8^+^ T cells (1.5 × 10^7^) freshly isolated or activated with anti-CD3/28 antibodies for 24 h according to the manufacturer's standard protocol (Active Motif, Carlsbad, CA, USA). Anti-JNK (#9258; Cell Signaling Technology, Danvers, MA, USA), anti-NFκB p65 (#8242; Cell Signaling Technology, Danvers, MA, USA) and rabbit IgG (Sigma-Aldrich, St Louis, MO, USA) as control antibody were used for the immunoprecipitation of chromatin. Immunoprecipitated DNA was analysed by RT–PCR with the following primer sets for NFκB p65: 5′-TTTGTCTGTTTCTCCTGCCTAC-3′ and 5′-GTGCGAATTACAGAATGGAAACC-3′. Ten primer sets for JNK/c-Jun (JNK1–JNK10) were shown in Supplementary Table 1. Data were presented as relative binding based on normalization to input DNA.

### Quantitative real-time PCR

Total RNA was extracted from cells using the RNeasy kit (Qiagen, Valencia, CA, USA) and retrotranscribed into cDNA using ABI Prism TaqMan reverse transcription reagents (Cat#204054, Applied Biosystems, Foster City, CA, USA). Specific primers of five NOX subsets for qRT–PCR were obtained from Invitrogen and the sequences were shown in Supplementary Tab. 2. Primers of CD39 were provided by Qiagen (NM_001164179, QT00081473). qRT–PCR was then performed using Quanti Fast SYBR Green PCR kit (Cat#204054, Qiagen, Valencia, CA, USA) on a Stratagene Fast Real Time Machine (Mx3005P) (Aligent Technologies, Santa Clara, CA, USA). Relative expression was calculated using the ΔΔCt algorithm and GAPDH as an internal control.

### Flow cytometric analysis

The relevant fluorescein-labelled anti-CD8, CD45 or CD3 antibodies were used to set-up the required compensation for flow cytometric analyses, with fluorescein-labelled isotype IgG used as a negative control in all cases for gating. For surface marker analyses, cells were stained with antibodies diluted in PBS containing 0.1% BSA^17^,^59^. To exclude dead cells, 4′,6-diamidino-2-phenylindole (DAPI, 3 μM) staining was used right after surface labelling. Lamina propria cells were gated with respect to the CD3^+^CD8^+^ population. For intracellular cytokine staining, cells were treated for 3 h with phorbol 12-myristate 13-acetate (50 ng ml^−1^), ionomycin (500 ng ml^−1^) and brefeldin A (10 μg ml^−1^). After surface staining, cells were washed by PBS, fixed by 2% paraformaldehyde and permeabilized with 0.5% saponin in PBS, followed by incubation with fluorescein-conjugated antibodies^17^,^59^. Fluorescence-activated cell sorting data were acquired on a multicolour LSRII (BD Biosciences, San Jose, CA, USA) and analysed with FlowJo software (TreeStar Inc., Ashland, OR, USA).

### Small hairpin RNA

Healthy CD8^+^ T cells were infected separately with an empty shRNA vector control (sh-C, pLKO.1-puro) or four different human NOX2 shRNA (sh-1: NM-000397.2-93s1c1, TRCN0000064590; sh-2: NM-000397.2-399s1c1, TRCN0000064588; sh-3: NM-000397.2-813s1c1, TRCN0000064591; sh-4: NM-000397.2-1637s1c1, TRCN0000064589) lentiviral transduction particles (Sigma-Aldrich, St Louis, MO, USA), according to the manufacturer's instructions. Recombinant lentiviral particles were produced by transient transfection of 293FT cells as described^60^. CD8^+^ T cells were infected with lentiviral particles^22^,^61^. Briefly, after stimulation with anti-CD3/28 antibodies (both 2 μg ml^−1^) and human recombinant IL-2 (R&D Systems, Minneapolis, MN, USA; 1 ng ml^−1^) for 24 h, CD8^+^ T cells were infected by lentiviral particles by centrifugation at 2,300 r.p.m for 60 min at room temperature in the presence of polybrene (8 μg ml^−1^). These cells were replaced with fresh media containing human recombinant IL-2 (R&D Systems, Minneapolis, MN, USA; 1 ng ml^−1^) for additional 48 h, and then selected with puromycin (1 μg ml^−1^) for 7–10 days. The survival cells were selected using dead cell removal kit (Miltenyi Biotec, San Diego, CA, USA). The selected cells were tested to confirm diminished NOX2 expression by western blot analysis.

### Reactive oxygen species detection

The cells were pretreated with 4 μM carboxy-2′,7′-dichlorodihydrofluorescein diacetate (H2DCFDA; Invitrogen, Carlsbad, CA, USA) with or without 10 μM DPI (Sigma-Aldrich, St Louis, MO, USA) or 10 μM VAS2870 (EMD Millipore, Darmstadt, Germany) for 5 min, then further stimulated with coated anti-CD3 antibody and soluble anti-CD28 antibody for indicated time. ROS generation was determined by flow cytometric analysis or microscope. The H2DCFDA-unstained cells were designated as the negative control.

### Ectonucleotidase ecto-enzymatic activity analysis

Phosphohydrolysis of extracellular nucleotides was analysed by thin layer chromatography (TLC), as established in our laboratory^17^,^62^. Sorted CD39^+^ or CD39^−^ CD8^+^ T cells (2 × 10^5^) were freshly used or stimulated with anti-CD3/CD28 antibodies for 3 h, and further incubated with 2 mCi ml^−1^ [C^14^]ADP (GE Healthcare Life Sciences, Pittsburgh, PA, USA) in 10 mM Ca^2+^ and 5 mM Mg^2+^ for indicated times. A volume of 5 μl of aliquots were removed at each time point and analysed for the presence of [C^14^]ADP hydrolysis products by TLC. [C^14^]ADP, [C^14^]AMP and [C^14^]ADO incubated in PBS served as standards. The TLC glass plate was exposed in a storage phosphor cassette for 24 h and phosphor release captured by a Strom Scanner. Adenosine uptake by cells was blocked with channel blocker dipyridamole at 10 μM.

### Statistical analysis

Results in this study are generally expressed as mean values±s.e.m. Differences between experimental groups were assessed by one-way analysis of variance. The two-tailed Student's *t-*test was used to compare two groups and the Tukey–Kramer multiple-comparison test was for multiple groups. Significance was defined as *P*<0.05.

## Additional information

**How to cite this article:** Bai, A. *et al.* NADH oxidase-dependent CD39 expression by CD8^+^ T cells modulates interferonγ responses via generation of adenosine. *Nat. Commun.* 6:8819 doi: 10.1038/ncomms9819 (2015).

## Supplementary Material

Supplementary Information Supplementary Figures 1-13 and Supplementary Tables 1-2
