# Author Correction: NADH oxidase-dependent CD39 expression by CD8^+^ T cells modulates interferon gamma responses via generation of adenosine

**DOI:** 10.1038/s41467-020-16314-5

**Published:** 2020-06-10

**Authors:** Aiping Bai, Alan Moss, Sonja Rothweiler, Maria Serena Longhi, Yan Wu, Wolfgang G. Junger, Simon C. Robson

**Affiliations:** 1 Division of Gastroenterology, Department of Medicine, Beth Israel Deaconess Medical Center, Harvard University, Boston, MA 02215 USA; 2 Department of Surgery, Beth Israel Deaconess Medical Center, Harvard University, Boston, MA 02215 USA

Correction to: *Nature Communications* 10.1038/ncomms9819, published online 9 November 2015.

In the original version of this Article, Fig. 3, panel a contained several errors.

1. The representative dot plot of the untreated cells, -DPI (leftmost image, upper row) has been inadvertently replaced with a duplicate image representing untreated cells, +DPI (leftmost dot plot from the lower row).

2. The frequency of gated cells in the samples treated with αCD3 and αCD28 Abs, -DPI (second and third dot plots, the upper row) was incorrectly labelled as 7.8 and 5.7 instead of 6.8 and 5.8, respectively.

3. The dot plots representing samples treated with αCD3 and αCD28 Abs, +DPI (second and third dot plots, the lower row) have been inadvertently switched. The correct version of Fig. 3a is:



The incorrect version is:



In addition, the upper dot plot in Fig. 3, panel e had been inadvertently replaced with a duplicate image from Fig. 3d. The correct version of Fig. 3e is:



The incorrect version is:



In the legend of Fig. 3e, the number of samples for CD226 expression is stated incorrectly as (*n* = 11). The correct number is (*n* = 18).

These errors are not corrected in the PDF or HTML versions of the Article.

