# Supplementary material for: NADH oxidase-dependent CD39 expression by CD8+ T cells modulates interferon gamma responses via generation of adenosine
Source: Nat Commun. 2015 Nov 9;6:8819. doi: 10.1038/ncomms9819 (PMC4667632; doi:10.1038/ncomms9819)
Supplement: Supplementary Information — Supplementary Figures 1-13 and Supplementary Tables 1-2 [file ncomms9819-s1.pdf]

Supplementary Figures and Legends

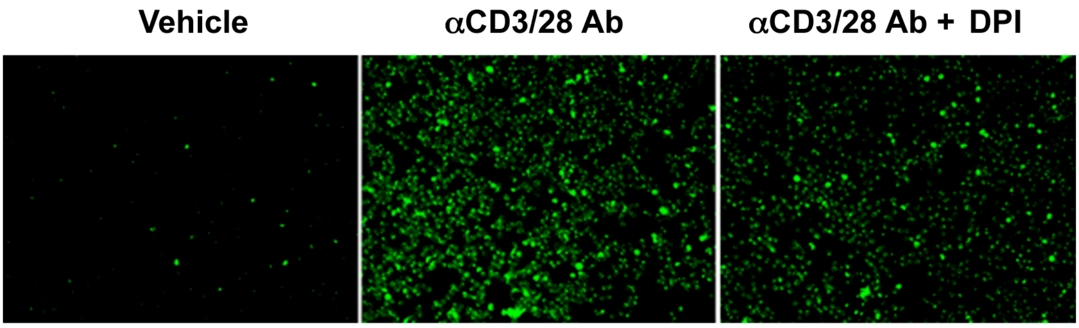

Supplementary Figure 1. NOX inhibition effectively blocks ROS generation. Healthy blood $CD8^+$ T cells were stimulated with anti-CD3/CD28 antibodies in the presence or absence of DPI (10 μM) for 1 hr, ROS induction was determined by microscopy (X 200 magnification).

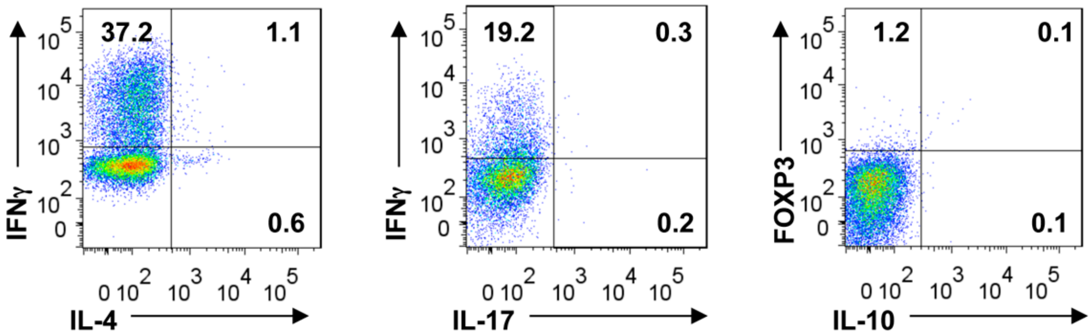

Supplementary Figure 2. Healthy blood CD8$^+$ T cells preferentially express IFNγ. Blood CD8$^+$ T cells were stimulated with anti CD3/28 antibodies for 24hrs, and intracellular staining of IFNγ, IL-4, IL-17, IL-10, or FOXP3, was analyzed by FACS. Data are representative of 3-4 independent experiments.

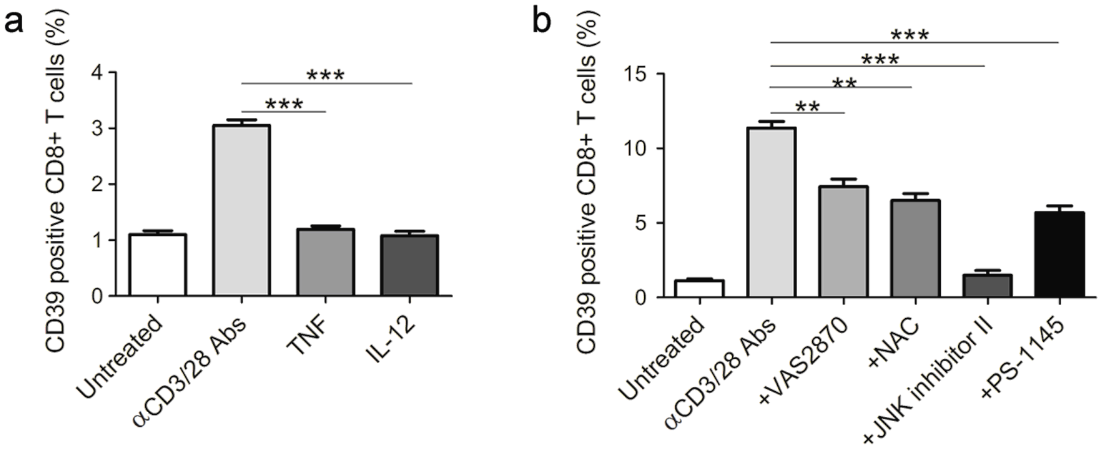

Supplementary Figure 3. Summary of FACS analyses of CD39 positive $CD8^+$ T cells. (a) Blood $CD8^+$ T cells were treated with anti-CD3 (10 μg/ml, precoated) and anti-CD28 (5 μg/ml, soluble) antibodies, or/and various reagents, as shown in Figure 2. CD39 expression was determined by flow cytometry at 24 hr (**a**) (n=4), or at 72 hr (b) (n=3). Data are presented as means ± S.E.M., $^{**}P<0.01$, $^{***}P<0.001$ (one-way ANOVA), with comparisons between groups.

```
                                                                  TTGAACCTGGGAGGCAGAGGTTGCAGTGAGCCAAGATCACGCCACTGCACT   -1951

                   c-Jun1                                                                                                  c-Jun2
-1950  CCAGCCTGGATGACAGAGTGAGGCTCTGTCTCAAAAAAAAGAATGTAAGAACGTAGTAAAATAGGCCAGGCATGGTGGCTCACGCCTGTAATCCCAGCACTTTGGGAGGCTGAGGCGGGCGGATCACCTGAGGTCAGGAGTTCGAGACCA   -1801

          c-Jun3
-1800  GCCTGACCAACATGGTGAAACCCCATCTCTACTAAAAATACACAAATTAGCTGGGCAAGGTGGCGGGCGCCTGTAATCCCAGCTACTTGGAGGCAGAGGCAGGAGAATCACTTGAACCCGGGAGGCAGAGGGTGCAGTAAGCTGAGATGG   -1651

                                                                                                                                           c-Jun4
-1650  CTCCATTGCACTCCAGCCTGGGAGACAAGAGTAAAACTCTTGTCTCAAAAAAAAAAAAAAAAAAAAGAACCTAATATCCTAATGTAATAAAGTAGAGAACCGTTGCCACAGCACCCACCAAAGTGTGTCTCTCATTGACAAGCTCAGGTAC   -1501

                                                 NFkB
-1500  TTGTTTGTCTGTTTCTCCTGCCTACCCCTCAGCCACTCTGGATCTTCCCTTTGATCATTCTTTTCTCTTTCTAGCTTTGATGTTTTGGTTTCCATTCTGTAATTCGCACACTATTTTGTGGACATGCTCTTTTCAAATTTCTCTCCAAAA   -1351

                     c-Jun5                                                                                                                 c-Jun6
-1350  CAATAGTACAAAGGCAGTCATAAACCAATTGTTGGTTTTTACAAAAAATCCCATCATATCACCAAAAACCAGTTTTATCATAACAGGATGAATATCAGACCAATATTCATTATTAGAGATGTAGTCTAAACTGACTCGGGAGAAGACATC   -1201

-1200  CATTGAGTCTTTCATTCAGTGTAATTAGTTGTTATCATACATGCAAAATCCTATAACTATAAAGCATTTCAGAAATAAAGTATTGTTAAACAGCTCCAAATTTATTAAAGTATAAAAAATGTATTGAGTATATACCATGTGGCACCGTGC   -1051

                                                           c-Jun7                       c-Jun8
-1050  AAAGTAACAGAGATGCCTAAACTAAAAGGTTATCCTTTGTCCTTATTAAAGTATGACTGAAAGCTCGCTGGAGAATATAGTTTCTACATAGAGTCAAAACAGTCCTTCGGGCTCACAAATTTAATGATTTATATGGTATAACTGGCTTTA   -901

 -900  GATACAAATTTACATGCCAGACAAGCAATGTAGAAAAATCAAGAACTAGGAAACAGGAAAACAGATCTACATTTCTGCTTTGCTGCAATTCATTCATTTTTTTCAACCATTCATCAAATATTTATTGAGAGCCCATGCTGTGAGAGAAAC   -751

 -750  TGTATTAGGTGCTGGGGAACATGGTAGCAAATAAGAGAGACCATGTATGTGAGCATGAGCAGGTGGCTTAACCTCTCCACATCTCTAACCTCTTTTAGACTCAATGTCTGAGGTATCTTTCAGACCTGAAATGCTAAGGTTTGCAACAGC   -601

                                                                                                                      c-Jun9
 -600  CTTGTGGAGGGAGAAGCTGCCAAGTCGCCTTGGGACTTGAGGACTTACCTTCCTTTCAAAGGATTAGCCCTTGTTTTGATTTCAAAATAATTTTGTTACACAGTTTAAAAATAATTTGGTCAGGCTCTCATCTACATTGGGGTAATCTTG   -451

       c-Jun10                                c-Jun11                c-Jun12
 -450  ACGGTCTGGATGTGGTAATTCATGCTCATTAGACTTCAAAGGTAGGAAGTCAAAGTTGGGAAATGGTGGATGACTTTCATCAATTCATAGCCAGGCAGCGGTTCCCATTGAGCAGCTCCTCTGTGGTTCCACCCAGCCTTCCAACCAATA   -301

                                  c-Jun13                                  c-Jun14                                c-Jun15
 -300  ACGCAGCGTCTCCTGCAGGAAACTCATCGCTTGGGTCACCTGTTGCTCTTTGCTCTAATGAGCCTTGAGAAAGGATTGCTGGTCATGGGACCAGAGGCTTTATGGGGAGGGAAGAACTGTTCTTGACTTTCAGTTTTTCGAGCGGGTTTC   -151

                                                                         c-Jun16
 -150  AAGGTAACAACTTTCTTATTATCTAGCTTCATAATTGCAGTAAGGAATAGCTTTTTTTGCTTGCAGCTGAGATGACTTTTTCAAGGGAGAAAAAGGGATTTCTATAACGAAGAGAGGGAGAGAGAGATGAAGAGGGAGGGAGTAAGGGAG   -1
```

Supplementary Figure 4. Putative sixteen JNK/c-Jun binding sites (c-Jun1 to c-Jun16, in red) and one of the NFκB p65-binding sites (NFκB, in blue) within the CD39 promoter region.

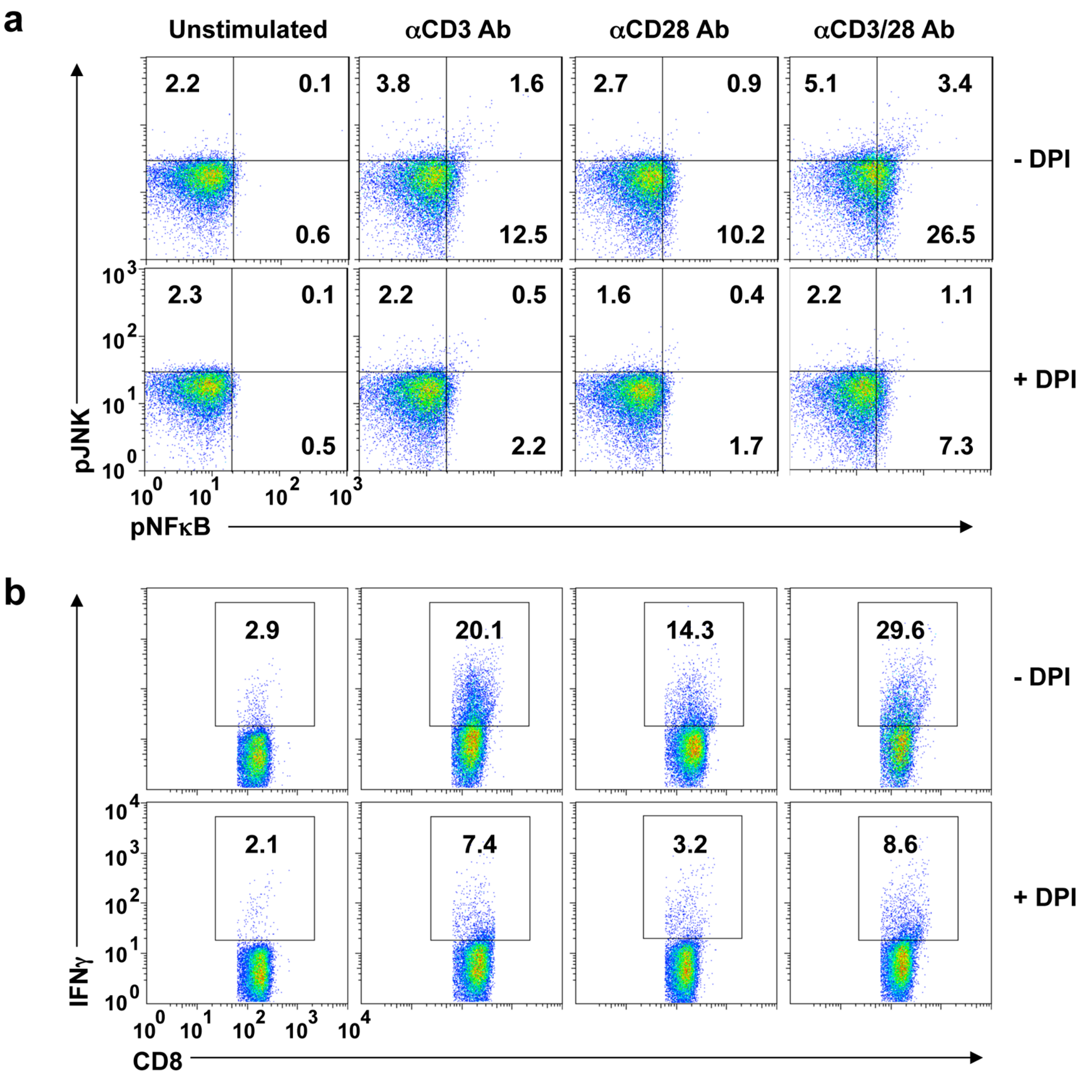

Supplementary Figure 5. Synergy between CD3 and CD28 signaling in Tc1 development. (**a**,**b**) Healthy blood CD8$^+$ T cells were stimulated with anti CD3 (10 μg/ml) or/and CD28 (5 μg/ml) antibodies in the presence of vehicle or DPI (10 μM), and intracellular staining of p-JNK and p-NFκB p65 was analyzed by at 60 min (**a**), or intracellular IFNγ expression was examined at 24 hr (**b**) by FACS. Data are representative of 3 independent experiments.

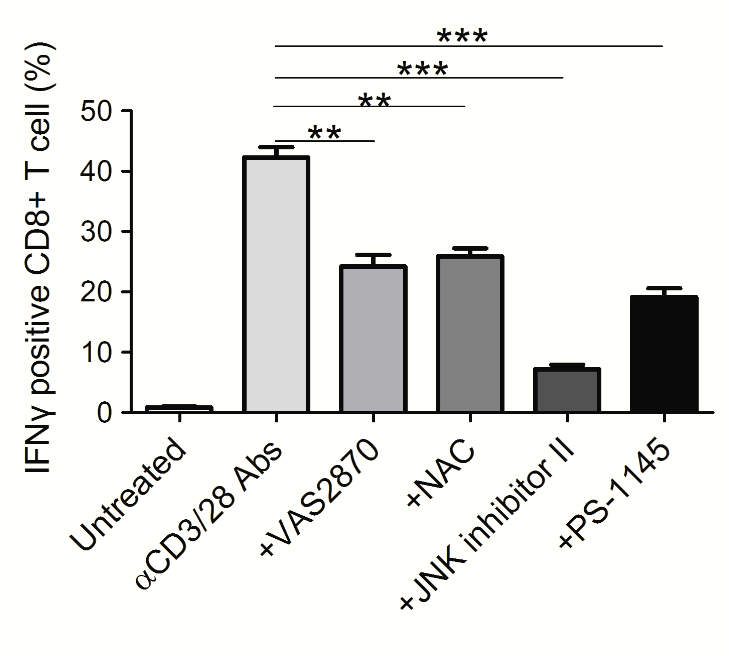

Supplementary Figure 6. Representative flow cytometry of IFNγ positive $CD8^+$ T cells. Blood $CD8^+$ T cells were stimulated with anti-CD3 (10 μg/ml, precoated) and anti-CD28 (5 μg/ml, soluble) antibodies, in the presence or absence of VAS2870 (10 μM), NAC (10 mM), JNK inhibitor II (10 μM), and PS-1145 (10 μM) for 24 hr (n=3). Data are presented as means ± S.E.M., ***P*<0.01, ****P*<0.001 (one-way ANOVA), with comparisons between groups.

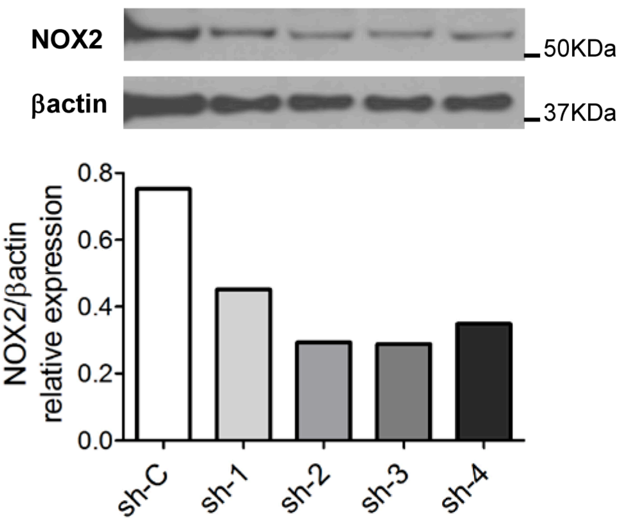

Supplementary Figure 7. Knockdown of NOX2 in CD8$^+$ T cells. Western blot analyses of NOX2 expression in healthy blood CD8$^+$ T cells after knock down using four lentiviral shRNAs (sh-1, 2, 3, and 4) and empty shRNA vector control (sh-C).

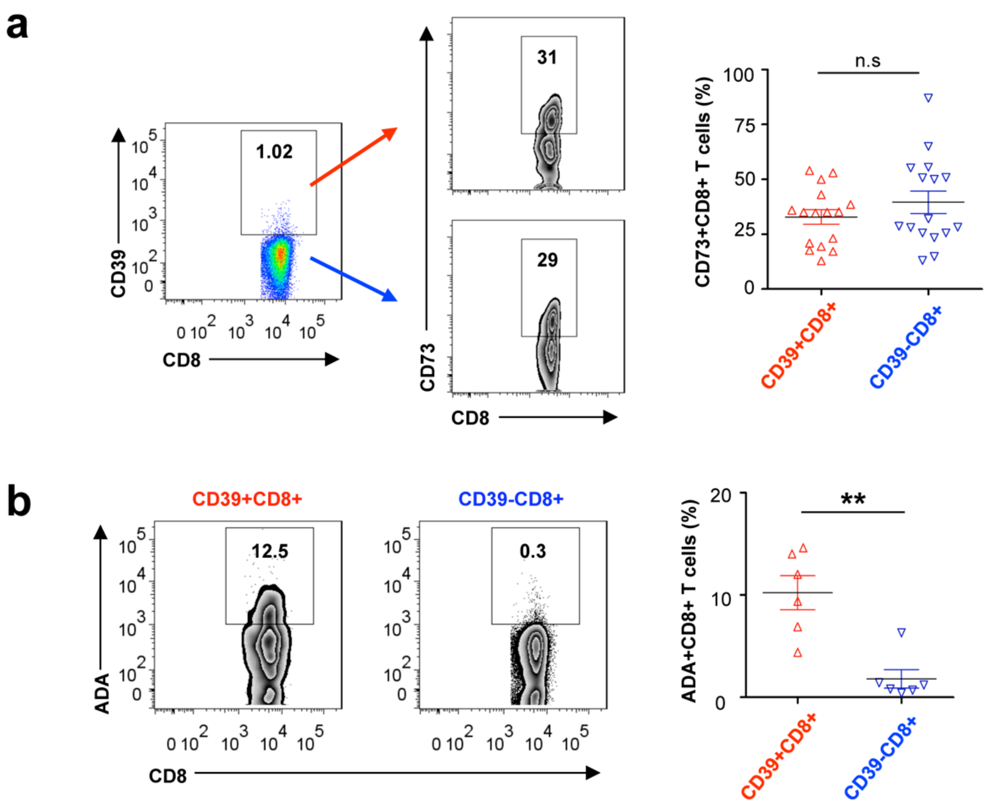

Supplementary Figure 8. Expression of CD73 and ADA in blood CD8$^+$ T cells. (**a**,**b**) Representative flow cytometric analyses of CD73 (**a**) and ADA (**b**) expression on blood CD39$^+$ and CD39$^-$ CD8$^+$ T cells. Statistical analyses of percentages of specific CD8$^+$ T cell subsets are shown on the right in each panel. Data are presented as means ± S.E.M., n.s.= non-significant. **P<0.001 (Student's t-test).

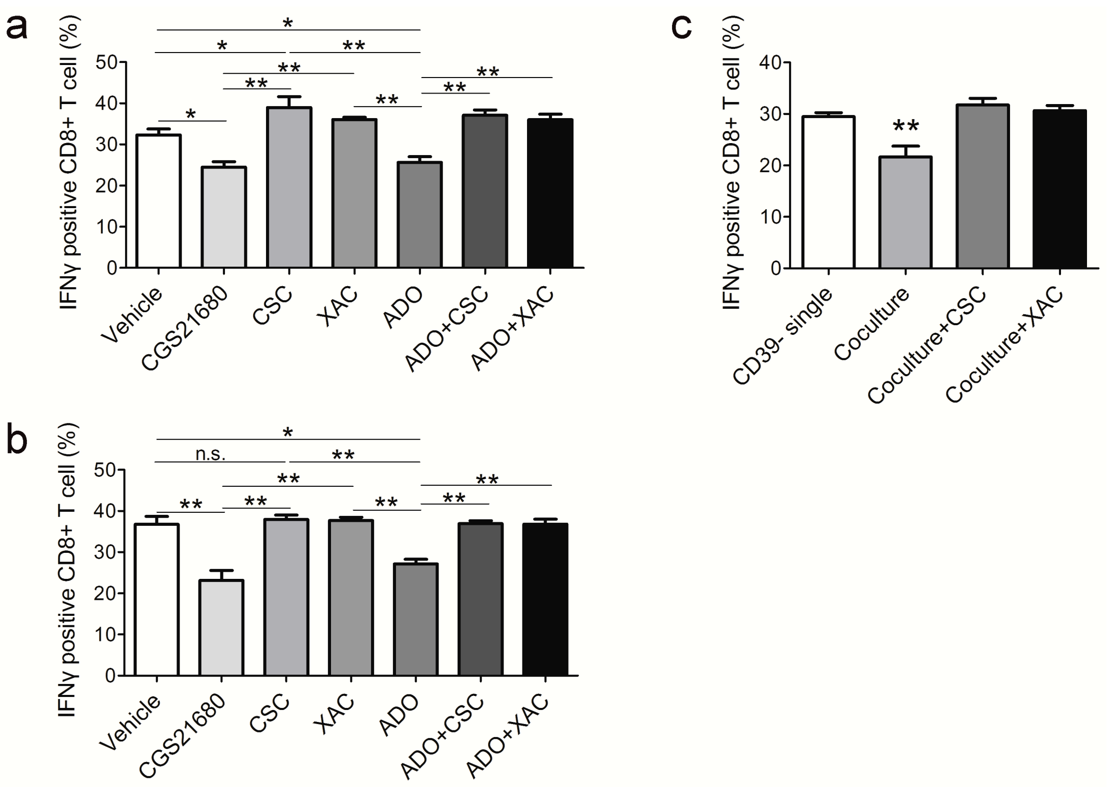

Supplementary Figure 9. Purinergic signaling modulates IFNγ expression by CD8$^+$ T cells. (**a,b**) Total CD8$^+$ T cells (**a**) or freshly sorted CD39$^-$CD8$^+$ T cells (**b**) were stimulated with anti-CD3/CD28 antibodies in the presence of vehicle or CGS21680 (100 nM), adenosine (ADO, 50 μM) alone or together with CSC (500 nM) or XAC (1 μM) for 24 hr, IFNγ expression was analyzed by FACS (n=3). Data are presented as mean ± S.E.M., n.s.= non-significant, *P<0.05, **P<0.01 (one-way ANOVA), with comparisons between groups. (**c**) CD39$^-$CD8$^+$ T cells (CFSE-labeled) alone or co-cultured with CD4$^+$CD39$^+$CD161$^+$ T cells were stimulated with anti-CD3/CD28 antibodies for 24 hr followed by FACS analysis of IFNγ expression. Co-cultures were incubated in the presence of vehicle, CSC (500 nM), or XAC (1 μM) (n=3). Data are presented as means ± S.E.M., **P<0.01 (one-way ANOVA), in comparison with the other groups.

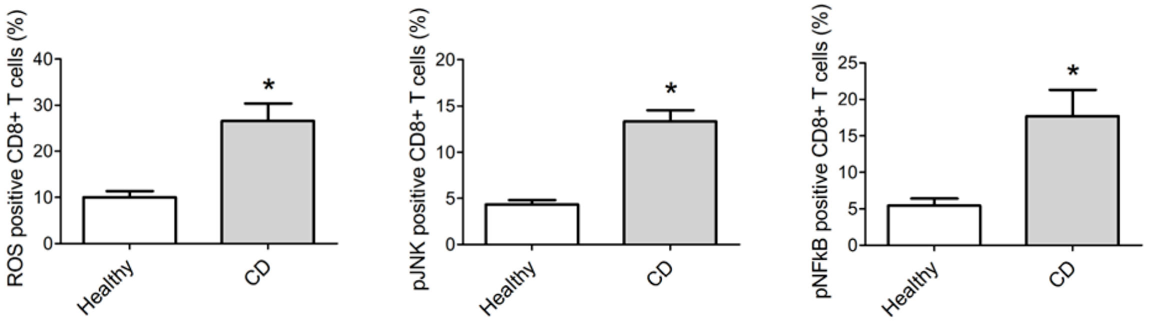

Supplementary Figure 10. Summary of FACS analyses of ROS induction (n = 4 and 7, for healthy and CD, respectively), intracellular pJNK (n = 4, 6) and pNFkB (n = 4, 6) levels in lamina propria $CD8^+$ T cells of healthy donors and active Crohn's disease (CD) patients. Data are presented as mean ± S.E.M., *P<0.05 (Student's t-test).

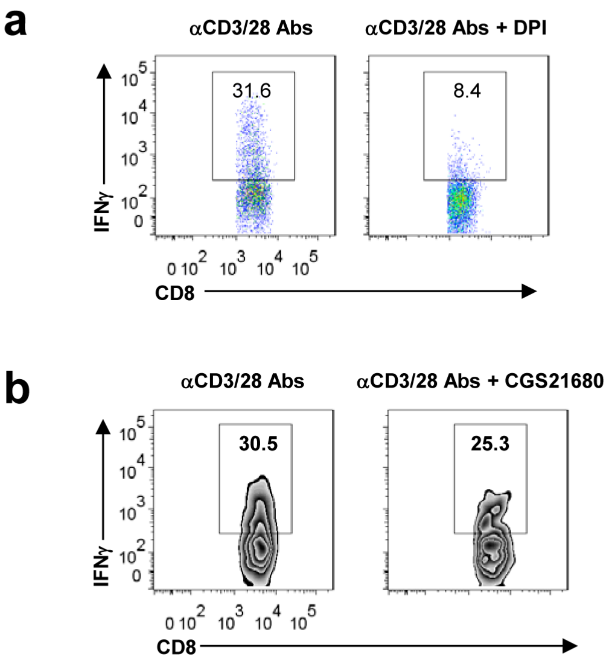

Supplementary Figure 11. Reactive oxygen species (ROS) and A2A signaling modulates Tc1 responses in lamina propria of Crohn's disease (CD). (**a,b**) Lamina propria CD8$^+$ T cells isolated from patients with active CD were stimulated with anti-CD3/CD28 antibodies in the presence of vehicle, DPI (10 μM) (**a**), or CGS21680 (100 nM) (**b**), respectively, for 24hr, followed by determination of intracellular IFNγ levels by FACS. Data are representative of 7 (**a**) or 3 (**b**) independent experiments.

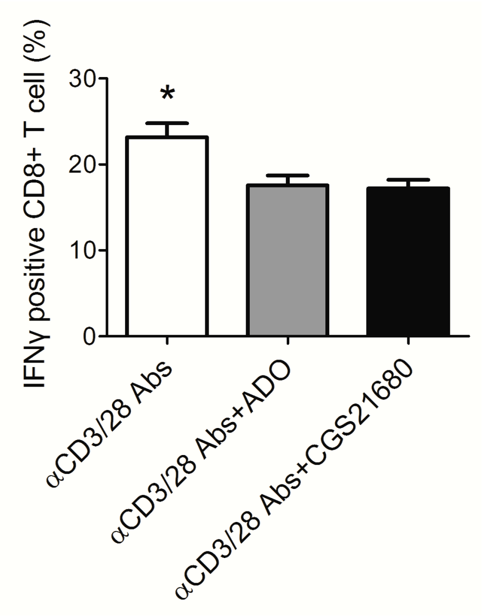

Supplementary Figure 12. A2A receptor agonists decrease IFNγ production of blood $CD8^+$ T cells from Crohn's disease (CD). Peripheral blood $CD8^+$ T cells of active CD patients were stimulated with anti-CD3/CD28 antibodies in the presence of adenosine (ADO, 50 μM), or CGS21680 (100 nM), followed by analyses of IFNγ expression at 24hr by FACS (n=3). Data are presented as mean ± S.E.M., *$P<0.05$ (one-way ANOVA), in comparison with other groups.

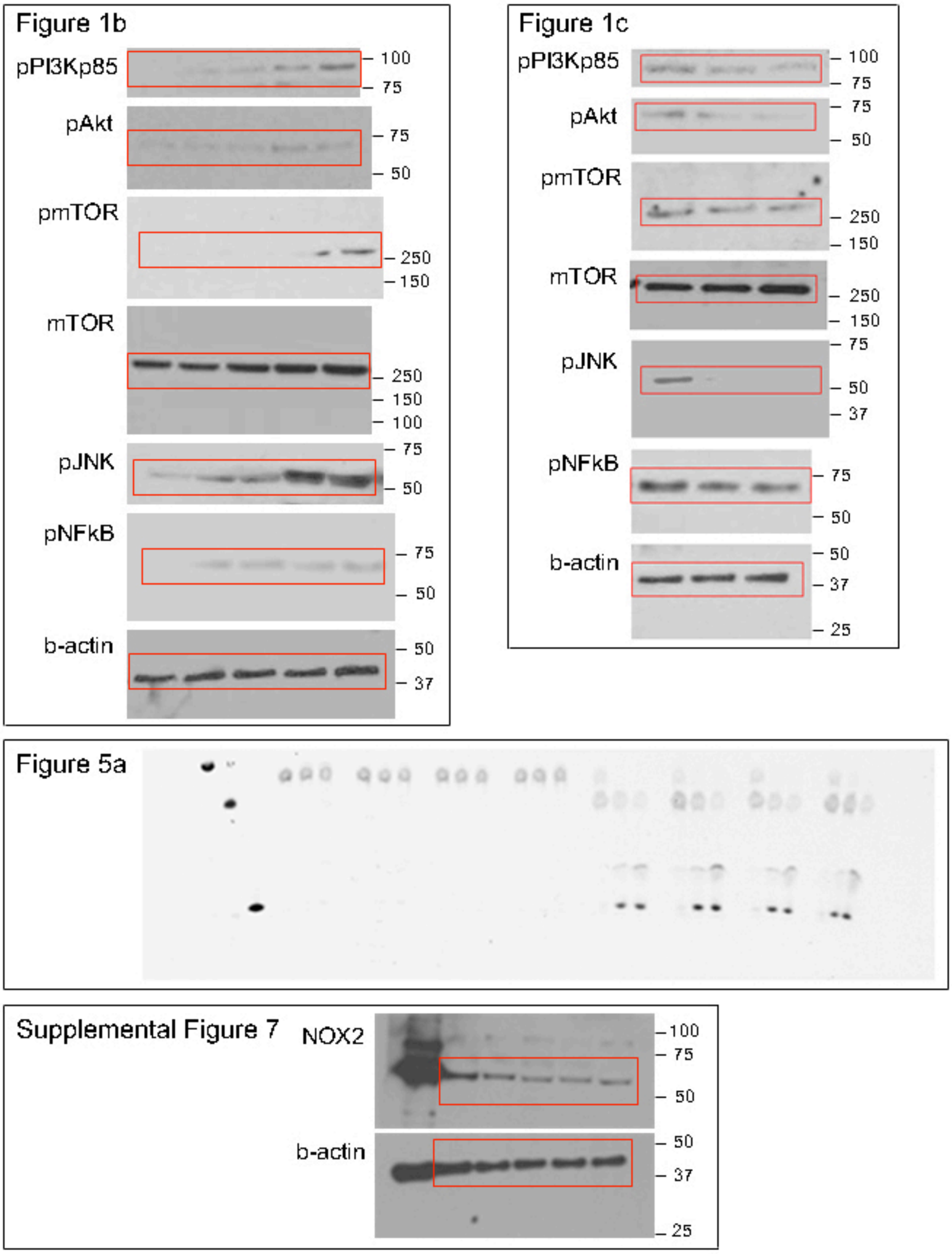

Supplementary Figure 13. The full blots of western shown and TLC in Figure 1b and c, Figure 5a, and Supplementary Figure 7.

Supplementary Table 1. Details the ten primer sets (from JNK1 to JNK10) used in the study.

Transcripts	Primer sequences	Positions
JNK1	Forward 5'- GGCAGAGGTTGCAGTGA -3'<br>Reverse 5'- CTGGGATTACAGGCGTGAG -3'	-1989 — -1854
JNK2	Forward 5'- CACTTTGGGAGGCTGAGG -3'<br>Reverse 5'- CCTCCAAGTAGCTGGGATTAC -3'	-1853 — -1707
JNK3	Forward 5'- CCATTCTGTAATTCGCACACTATTT -3'<br>Reverse 5'- TGGTCTGATATTCATCCTGTTATGA -3'	-1409 — -1248
JNK4	Forward 5'- TCATAACAGGATGAATATCAGACCA -3'<br>Reverse 5'- CACTGAATGAAAGACTCAATGGATG -3'	-1273 — -1180
JNK5	Forward 5'- ACCGTGCAAAGTAACAGAGAT -3'<br>Reverse 5'- CATTGCTTGTCTGGCATGTAAA -3'	-1057 — -871
JNK6	Forward 5'- ACTTGAGGACTTACCTTCCTTTC -3'<br>Reverse 5'- GTCTAATGAGCATGAATTACCACAT -3'	-566 — -417
JNK7	Forward 5'- TCATGCTCATTAGACTTCAAAGGTA -3'<br>Reverse 5'- CAGAGGAGCTGCTCAATGG -3'	-431 — -328
JNK8	Forward 5'- GGTTCCACCCAGCCTTC -3'<br>Reverse 5'- CTCATTAGAGCAAAGAGCAACAG -3'	-326 — -239
JNK9	Forward 5'- CCTGTTGCTCTTTGCTCTAATG -3'<br>Reverse 5'- AGAAAGTTGTTACCTTGAAACCC -3'	-362— -135
JNK10	Forward 5'- CGGGTTTCAAGGTAACAACTTTC -3'<br>Reverse 5'- CTCTCTCTCCCTCTCTTCGTTAT -3'	-157 — -25

Supplementary Table 2. qRT-PCR primers. This listing shows the primer sequences used in the study.

Transcript	Primer sequence
NOX1	Forward 5'- TGAGAGGACAGAGGCAAATAAC -3'
	Reverse 5'- GAGATGGAAGAGAAAGGGAGATG -3'
NOX2	Forward 5'- GCTATGAGGTGGTGATGTTAGT -3'
	Reverse 5'- CTTCAGATTGGTGGCGTTATTG -3'
NOX 3	Forward 5'- CTGAAGCTGAGCAAGGTGTAT -3'
	Reverse 5'- CGTGTTTCCAGGGAGAGTAAG -3'
NOX4	Forward 5'- GCAGCAAGATACCGAGATGAG -3'
	Reverse 5'- GAGGCTGTGATCATGAGGAATAG -3'
NOX5	Forward 5'- GGTCTGCTTCATCGCATTAGTA -3'
	Reverse 5'- CTCTCTGTTGAGCAGGATTAGG -3'
